# Características clínicas, histopatológicas e inmunohistoquímicas de pacientes con síndrome seco y biopsia de glándula salival con puntaje de foco de 1 o más

**DOI:** 10.7705/biomedica.7315

**Published:** 2025-03-28

**Authors:** Andrés Felipe Lamos-Duarte, Rafael Parra-Medina, Carlos Santiago Rivadeneira-Chamorro, Juan Pablo Castañeda-González, Alejandro Escobar, Adriana Rojas-Villarraga, Gabriel Santiago Rodríguez-Vargas, Ana María Arredondo, Héctor Cubides, José Fernando Polo, Juan José Capasso, Claudia Ibáñez, Jairo Hernán Cajamarca-Barón

**Affiliations:** 1 Departamento de Reumatología, Fundación Universitaria de Ciencias de la Salud - FUCS, Hospital de San José, Bogotá, D. C., Colombia Fundación Universitaria de Ciencias de la Salud - FUCS Fundación Universitaria de Ciencias de la Salud - FUCS Hospital de San José Bogotá, D. C. Colombia; 2 Laboratorio de Patología, Instituto Nacional de Cancerología y Fundación Universitaria de Ciencias de la Salud - FUCS, Bogotá, D. C., Colombia Instituto Nacional de Cancerología Instituto Nacional de Cancerología Fundación Universitaria de Ciencias de la Salud - FUCS Bogotá, D. C. Colombia; 3 Instituto de Investigaciones, Fundación Universitaria de Ciencias de la Salud - FUCS, Bogotá, D. C., Colombia Fundación Universitaria de Ciencias de la Salud - FUCS Fundación Universitaria de Ciencias de la Salud - FUCS Bogotá, D. C. Colombia; 4 Dirección Científica, Biomab I.P.S., Bogotá, D. C., Colombia Biomab I.P.S. Biomab I.P.S. Bogotá, D. C. Colombia; 5 Departamento de Patología, Hospital Infantil Universitario de San José, Fundación Universitaria de Ciencias de la Salud - FUCS, Bogotá, D. C., Colombia Hospital Infantil Universitario de San José Hospital Infantil Universitario de San José Fundación Universitaria de Ciencias de la Salud - FUCS Bogotá, D. C. Colombia; 6 Departamento de Patología, Hospital de San José, Fundación Universitaria de Ciencias de la Salud - FUCS, Bogotá D. C., Colombia Hospital de San José Hospital de San José Fundación Universitaria de Ciencias de la Salud - FUCS Bogotá D. C. Colombia

**Keywords:** síndrome de Sjögren, inmunohistoquímica, biopsia, linfocitos, enfermedades autoinmunes, Sjögren’s syndrome, immunohistochemistry, biopsy, lymphocytes, autoimmune diseases

## Abstract

**Introducción.:**

El síndrome de Sjögren es una enfermedad autoinmunitaria sistémica. Se ha descrito la utilidad de la inmunohistoquímica de la biopsia de glándula salival menor por su utilidad en la caracterización del fenotipo de los linfocitos en los casos de difícil diagnóstico.

**Objetivo.:**

Describir las variables sociodemográficas, clínicas, serológicas, histopatológicas e inmunohistoquímicas de los pacientes con síndrome seco en quienes se practicó biopsia de glándula salival menor que resultó con un puntaje de foco de uno o más.

**Materiales y métodos.:**

Se adelantó un estudio observacional, retrospectivo, en el que se incluyeron pacientes en estudio por posible síndrome seco y cuya biopsia de glándula salival menor estaba disponible y había obtenido un puntaje de foco de uno o más. En la biopsia de la glándula salival menor se realizó inmunohistoquímica con cromógeno rojo para la identificación de los linfocitos T CD8 positivos y marrón, para los linfocitos T CD4 positivos. Se determinó la relación de los marcadores CD20:CD3 y CD4:CD8 con el equipo MoticEasyScan Pro-6™ (MOTIC) y el *software* QuPath^™^. El análisis de las variables cualitativas se realizó mediante la aplicación de la prueba de ji al cuadrado (c^2^) o la prueba exacta de Fisher; para el de las variables cuantitativas, la prueba seleccionó según el supuesto de normalidad.

**Resultados.:**

Se analizaron 28 pacientes, 16 con síndrome de Sjögren y 8 con poliautoinmunidad. Se encontró una asociación entre la presencia de atrofia glandular en la biopsia de glándula salival menor y el desarrollo de poliautoinmunidad (OR = 11,1; IC _95%_: 1,12-112; p = 0,033). Las relaciones CD20:CD3 y CD4:CD8 fueron normales, sin diferencias estadísticamente significativas entre pacientes con síndrome de Sjögren y sin él. En el subgrupo de pacientes con síndrome de Sjögren hubo predominancia de los linfocitos T CD4, 15 de los 16 casos tenían una relación CD4:CD8 igual o mayor de 2:1.

**Conclusiones.:**

Se encontró una asociación entre la atrofia glandular y la presencia de poliautoinmunidad, así como predominancia de los linfocitos T CD4 en los pacientes con síndrome de Sjögren. Esto resalta el posible valor de aplicar la inmunohistoquímica en las biopsias de glándula salival menor en este grupo.

## Abstract (en)

**Introduction.:**

Sjögren’s syndrome is a systemic autoimmune disease. The usefulness of immunohistochemistry in minor salivary gland biopsies has been described to be helpful in indirectly characterizing the lymphocyte phenotype in difficult diagnosis cases.

**Objective.:**

To describe sociodemographic, clinical, serological, histopathological, and immunohistochemical variables in patients with sicca syndrome and a minor salivary gland biopsy focus score greater than or equal to one.

**Materials and methods.:**

We conducted an observational, retrospective study that included patients under study for potential sicca syndrome whose minor salivary gland biopsy was available and had obtained a focus score greater than or equal to one. Immunohistochemistry was performed on the minor salivary gland biopsy with chromogen red staining for CD8 T lymphocytes and brown staining for CD4 T lymphocytes. Expression ratio of CD20:CD3 and CD4:CD8 markers was determined with the MoticEasyScan Pro 6™ (MOTIC) device and the QuPath™ software. Qualitative variables were analyzed using the chi-square or Fisher’s exact test, and quantitative variables were analyzed according to their assumption of normality.

**Results.:**

Twenty-eight patients were analyzed: 16 patients had Sjögren’s syndrome, and 8 of them had polyautoimmunity. An association was found between atrophy in the minor salivary gland biopsy and development of polyautoimmunity (OR = 11.1; 95% CI: 1.12-112; p value = 0.033). The CD20:CD3 and CD4:CD8 ratios were normal, with no statistically significant differences between patients with and without Sjögren’s syndrome. In the subgroup of patients with Sjögren’s syndrome, CD4 T lymphocytes were predominant, with 15 cases out of 16 with CD4:CD8 ratios equal to or greater than 2:1.

**Conclusions.:**

Glandular atrophy was associated with the development of polyautoimmunity and a predominance of CD4 T lymphocytes in patients with Sjögren’s syndrome. This finding highlights the potential value of immunohistochemistry of minor salivary gland biopsies in this group.

El síndrome de Sjögren es una enfermedad autoinmunitaria sistémica de etiología multifactorial, caracterizada por la infiltración y activación aberrante de los linfocitos T y B, que afecta la función de la unidad secretora de las glándulas exocrinas ^1^^,^^2^.

Su incidencia global anual es de 7 casos por cada 100.000 personas y una prevalencia de 43 a 60 casos por cada 100.000 habitantes. Sin embargo, estas cifras dependen de los criterios clasificatorios y las condiciones sociodemográficas de las poblaciones estudiadas ^3^. Se estima que el síndrome de Sjögren ocupa el segundo lugar de prevalencia a nivel mundial entre las enfermedades autoinmunitarias sistémicas ^4^.

Los criterios del *American College of Rheumatology* (ACR) y la anteriormente llamada *European League against Rheumatism* (EULAR) del 2016 son los más recientes y ampliamente aceptados para la clasificación del síndrome de Sjögren ^5^. Estos incorporan una combinación de variables clínicas, serológicas e histopatológicas que incluyen la biopsia de la glándula salival menor con hallazgos específicos de sialoadenitis linfocítica focal asociada a un puntaje de foco de uno o más por cada 4 mm^2^ de tejido ^5^.

Respecto a estos criterios, es necesario cumplir con cuatro puntos, al menos, el puntaje de foco ≥ 1 y la presencia de anticuerpos anti-SSA/Ro son los de mayor importancia para clasificar esta enfermedad ^5^^,^^6^

Los pacientes con síndrome de Sjögren tienen un riesgo de 9 a 16 veces mayor de desarrollar neoplasias sólidas o linfoides. El linfoma no Hodgkin de la zona marginal es la neoplasia más frecuente en estos pacientes, donde la hipertrofia persistente de las glándulas salivales es el factor de riesgo clínico más importante para el desarrollo del síndrome de Sjögren ^7^^,^^8^.

En términos de síntomas clínicos y hallazgos histopatológicos, es un desafío discernir entre el subtipo de linfoma no Hodgkin de la zona marginal asociado a las mucosas y las lesiones inflamatorias relacionadas con el síndrome de Sjögren, ya que no existen marcadores específicos de inmunohistoquímica disponibles para su diagnóstico. Por esta razón, se han desarrollado varios ensayos de laboratorio para la detección de reordenamientos clonales de genes de inmunoglobulinas asociados al proceso de formación de linfomas. Estos se basan en la teoría de que los linfocitos comparten orígenes clonales ^9^^-^^11^.

Debido al alto costo de tales pruebas moleculares, con frecuencia se prefiere el uso de la inmunohistoquímica (sin ser una práctica rutinaria) para la identificación de clones de linfocitos aberrantes. Esta técnica permite caracterizar indirectamente el fenotipo de los linfocitos presentes en la biopsia de glándula salival menor, sobre todo en situaciones de difícil diagnóstico y en el análisis de muestras de pacientes incluidos en ensayos clínicos ^6^. La inmunohistoquímica es un método que utiliza anticuerpos dirigidos contra marcadores específicos de ciertos tejidos para determinar el tipo de célula y el órgano de origen ^12^.

Son escasos los estudios que describen la caracterización y la distribución de las células T y B asociadas a diversos factores clínicos en los pacientes con síndrome de Sjögren. Hasta la fecha no hay una estandarización inmunohistoquímica que permita la interpretación precisa de los hallazgos histopatológicos y del fenotipo linfocitario específico en biopsias de glándula salival menor de estos pacientes. Sin embargo, Fisher *et al*. ^6^ sugieren incluir tinciones para CD3, CD20 y CD21, determinar la presencia de estructuras similares a los centros germinales y analizar la proporción de focos con segregación de células T y B y de redes de células dendríticas foliculares dentro del estudio ampliado de la biopsia. Todo lo anterior podría mejorar la precisión diagnóstica de la biopsia de glándula salival menor en los pacientes con síndrome de Sjögren ^6^.

Así, pues, el objetivo de este estudio fue describir las variables sociodemográficas, clínicas, serológicas e histopatológicas de los pacientes con síndrome seco cuya biopsia de glándula salival menor tuvo un puntaje de foco de uno o más. Además, se caracterizaron las diferentes subpoblaciones linfocitarias mediante la aplicación de inmunohistoquímica con anticuerpos dirigidos contra los marcadores CD3, CD4, CD8 y CD20 en la biopsia de la glándula salival menor.

## Materiales y métodos

### Población

Se llevó a cabo un estudio observacional, retrospectivo, en el que se seleccionaron pacientes que cumplieran los siguientes criterios de inclusión:

1. mayores de 18 años;

2. sujetos con síndrome seco en estudio y en seguimiento por el Servicio de Reumatología del Hospital de San José (Bogotá, Colombia), desde enero del 2019 hasta diciembre del 2022, a quienes se les había solicitado biopsia de glándula salival menor como herramienta diagnóstica, luego de excluir antecedentes clínicos de otras enfermedades (infección por hepatitis C activa, sida, sarcoidosis, amiloidosis, enfermedad de injerto contra huésped y enfermedad relacionada con IgG4), según los lineamientos de ACR/EULAR 2016 ^5^, y

3. pacientes con disponibilidad de biopsia de glándula salival menor, cuyo resultado tuviera un puntaje de foco de uno o más, criterio usado por ACR/EULAR 2016 para clasificar el síndrome de Sjögren ^5^.

Un foco se definió como el acúmulo de 50 o más células mononucleares en un área de 4 mm^2^ de tejido de apariencia normal. El puntaje de foco se calculó como el número de focos por área de la muestra (mm^2^) multiplicado por cuatro. La técnica con la que se tomó la biopsia de glándula salival menor fue la convencional mediante incisión lineal horizontal única, de 5 mm a 10 mm, paralela al borde de la superficie interna del labio inferior a través de la mucosa ^6^^,^^13^.

Se recolectaron datos de variables sociodemográficas (sexo, edad, educación y seguridad social), clínicas (duración de la enfermedad, manifestaciones extraglandulares, comorbilidades y poliautoinmunidad definida como la presencia de dos o más enfermedades autoinmunitarias confirmadas) ^14^ y serológicas (anticuerpos antinucleares, anticuerpos nucleares extraíbles, hemograma y factor reumatoide).

Se clasificaron como síndrome de Sjögren aquellos casos que cumplían los criterios ACR/EULAR 2016 ^5^. En aquellos pacientes clasificados como síndrome de Sjögren, se calculó el índice de actividad de la enfermedad *EULAR Sjögren’s Syndrome Disease Activity Index* (ESSDAI).

### Características histopatológicas

Se recuperaron los bloques de parafina y las láminas de la biopsia de glándula salival menor del Servicio de Patología del hospital. Las láminas no disponibles en la institución se solicitaron al banco de almacenamiento nacional de tejidos. Posteriormente, se procedió a la digitalización de las láminas disponibles mediante el equipo MoticEasyScan Pro-6™ (MOTIC).

Cada muestra fue evaluada de forma independiente por un médico patólogo experto en la lectura de biopsias de glándula salival menor. Se describieron los hallazgos microscópicos, como área (mm^2^) de la glándula, número de lóbulos, inflamación linfocítica (leve, moderada, grave), número de focos, presencia de dilatación del conducto, fibrosis (fibras de colágeno alrededor de los conductos o acinos), atrofia (pérdida del parénquima glandular) y adiposis (reemplazo del parénquima por adipocitos); número de centros germinales (infiltrado circunscrito de linfocitos B, linfocitos T en menor cantidad, macrófagos con cuerpos tingibles y células dendríticas foliculares), presencia o ausencia de granulomas y cálculo del puntaje de foco (número de focos presentes en el área total de la glándula (mm^2^), multiplicado por cuatro). Se define un puntaje de foco positivo cuando el valor obtenido es igual o mayor a un foco por cada 4 mm^2^ de tejido glandular.

### Análisis de inmunohistoquímica

Se practicaron estudios de inmunohistoquímica y se analizaron en cada uno de los casos seleccionados previamente. Estos estudios se ejecutaron en técnica dual: luego de obtener nuevos cortes de las biopsias seleccionadas, se realizó la tinción con los cromógenos rojo y marrón. La técnica dual se empleó para permitir la comparación entre los marcadores CD20 y CD3, y CD4 y CD8. Los linfocitos inmunorreactivos para CD20 (linfocitos B) y CD8 (linfocitos T citotóxicos) se tiñeron de color rojo, mientras que los inmunorreactivos para CD3 (linfocitos T, en general) y CD4 (linfocitos T ayudadores) se tiñeron de color marrón. Los clones utilizados para cada anticuerpo fueron 2GV6 (CD3), SP35 (CD4), SP57 (CD8) y L26 (CD20). Todas las láminas tuvieron como control de tejido externo un fragmento de amígdala.

Finalmente, se digitalizaron las nuevas láminas por medio del MoticEasyScan Pro-6™ (MOTIC) para someter parte del análisis al software relacionado. Dicho análisis constó de dos partes: en la primera se revisó la marcación de las biopsias para definir la positividad de los linfocitos para cada uno de los marcadores y se determinaron los agregados linfoides más representativos; en la segunda, con la marcación preestablecida, se realizó un análisis cuantitativo mediante el *software* QuPath™ para estimar el número de linfocitos reactivos para cada marcador y determinar su relación según el inmunofenotipo expresado, utilizando una razón aritmética.

Posteriormente, con el *software* RedCap™ ^15^, se desarrolló un cuestionario de cinco módulos para la recolección de datos sociodemográficos, manifestaciones clínicas, datos paraclínicos, factores de riesgo para el desarrollo de linfoma y características histopatológicas de la biopsia de la glándula salival menor que incluía los análisis inmunohistoquímicos.

### Análisis estadístico

Se hizo un análisis descriptivo mediante frecuencias absolutas y relativas para las variables cualitativas. Para las variables cuantitativas, se utilizó la media y la desviación estándar (DE), o la mediana y el rango intercuartílico (RIC) en función de la normalidad de su distribución. Esta normalidad se evaluó por medio de las pruebas de Shapiro-Wilk y Kolmogorov-Smirnov, según la naturaleza de las variables.

Para evaluar la diferencia entre las frecuencias de las manifestaciones clínicas, paraclínicas e inmunohistoquímicas, se utilizó la prueba de ji al cuadrado (c^2^) mediante el método de Pearson o Fisher, según el caso. Para explorar la asociación entre la presencia de poliautoinmunidad y atrofia glandular se calculó la razón de momios (*odds ratio*, OR) con su respectivo intervalo de confianza del 95 %. Se consideró significativo un valor de p menor de 0,05 (p < 0,05). El análisis de datos se ejecutó en el *software* SPSS™, versión 23.

### Consideraciones éticas

El estudio fue aprobado por el Comité de Ética en Investigación con Seres Humanos (CEISH) del Hospital de San José, mediante el Acta 486 del 2022.

## Resultados

Se incluyeron inicialmente 42 pacientes con síntomas de síndrome seco y con resultados de *focus score* mayor o igual a uno en la biopsia de glándula salival menor. Se contó con la historia clínica de 31 pacientes, de las cuales se pudieron analizar 28 que tenían completos los datos clínicos. La edad promedio fue de 51,26 ± 15 años. Todos los pacientes fueron de sexo femenino y 20 estaban afiliadas al régimen contributivo del sistema de seguridad social del país. Respecto a las comorbilidades, 11 casos presentaban hipotiroidismo, las demás se reportan en el cuadro 1.

**Figura 1:**
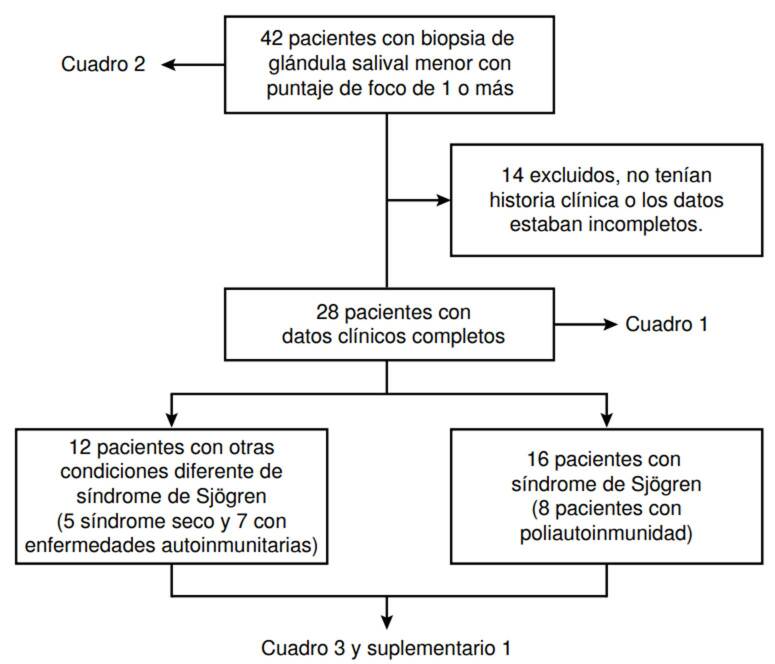
Selección de pacientes

Por otro lado, del total de pacientes, 16 tenían síndrome de Sjögren según los criterios definidos, 5 tenían síndrome seco sin enfermedad definida y 7 presentaban enfermedad reumatológica autoinmunitaria sistémica. De las pacientes que presentaban síndrome de Sjögren, 8 tenían poliautoinmunidad; 3 padecían lupus eritematoso sistémico; una, enfermedad mixta del tejido conjuntivo; 2, artritis reumatoide; una, esclerosis sistémica, y una, miopatía inflamatoria idiopática (figura 1 y cuadro 1).

**Cuadro 1:** Características de la población incluida con resultado de puntaje de foco positivo (N = 28)

Variables		n	
Sexo femenino		28	
Edad (años), media (DE)		51,2	
Educación, n		14	
	Primaria	3	
	Secundaria	5	
	Tecnológica	4	
	Profesional	2	
Seguridad social, n			
	Régimen contributivo	20	
Pacientes con síndrome de Sjögren, n		16	
	Enfermedad única	8	
	Poliautoinmunidad*	8	
Duración de síntomas secos (años), mediana (RIC)		2	(3)
Manifestaciones sistémicas, n		15	
	Articular	5	
	Hematológica	3	
	Pulmonar	3	
	Del sistema nervioso periférico	3	
	Cutánea	1	
Otras manifestaciones, n			
	Xeroftalmia	6	
Estudios serológicos, n			
	ANA positivos	26	
	1:40 - 1:160	13	
	1:320 - 1:2560	13	
Patrones ANA, n		23	
	Granular	11	
	Homogéneo	6	
	Centromérico	4	
	Citoplasmático	2	
ENA positivos, n		25	
	Anti-ro positivos	12	
	21 a 80 U/L	5	
	81 a 160 U/L	6	
	161 a 320 U/L	1	
Factor reumatoide, mediana (RIC)		30,5	(77)
	Positivo (> 20 UI/ml)	16	
Hemograma, n			
	Leucopenia	2	
	Anemia	3	
Comorbilidades, n			
	Diabetes mellitus	16	
	Hipotiroidismo	11	
	Hipertensión arterial	6	
	Dislipidemia	3	
	Hipertensión pulmonar	3	
	Síndrome de apnea e hipopnea del sueño	2	
	Enfermedad pulmonar obstructiva crónica	1	
	Falla cardíaca	1	

DE: desviación estándar; RIC: rango intercuartílico; ANA: anticuerpos antinucleares; ENA: anticuerpos nucleares extraíbles

* Poliautoinmunidad de síndrome de Sjögren con lupus eritematoso (n=3), artritis reumatoide (n=2), esclerosis sistémica (n=1), miopatía inflamatoria idiopática (n=1) y enfermedad mixta del tejido conjuntivo (n=1)

La mediana de duración de los síntomas secos fue de dos años (RIC = 1-3) y además de estos síntomas, 15 de las pacientes presentaron compromiso articular, leve en 5 casos. En cuanto a las características serológicas, 11 casos presentaron un patrón granular fino denso de ANA (clasificación AC-4) y la mitad presentó títulos mayores o iguales a 1:320. Los anticuerpos anti-ro, se presentaron en 12 casos del total estudiado, en su mayoría con títulos menores o iguales a 1:160. En el subgrupo de los 16 casos con síndrome de Sjögren, 9 expresaron estos anticuerpos (prueba anti-ro positiva). El factor reumatoide fue positivo en 16 pacientes, con la misma proporción en el subgrupo de pacientes con síndrome de Sjögren confirmado. Solo dos pacientes presentaron leucopenia y tres, anemia, ambas leves (cuadro 1). Un paciente registró agrandamiento persistente de la glándula parótida, mientras que en 20 pacientes se reportó consumo de la proteína C3 del sistema complemento y en 19, consumo de la C4. No se encontró ningún caso con linfoma no Hodgkin asociado a las mucosas y no se reportaron pacientes con crioglobulinas.

En el cuadro 2 se presentan los hallazgos histopatológicos de las biopsias de las glándulas salivales menores del total de los pacientes con síntomas secos y puntaje de foco de uno o más, independientemente de la disponibilidad de los datos de la historia clínica. En 29 casos se presentó inflamación moderada y 16 pacientes presentaron dilatación del conducto, fibrosis y atrofia. En ocho casos se encontró adiposis y solo en un paciente se documentó la presencia de centros germinales. No se encontraron granulomas en las biopsias de las glándulas salivales menores analizadas. Para el puntaje de foco se determinó una mediana de 1,4 (RIC = 0,7), 36 casos tuvieron un puntaje de foco de 2 o más. Además, se encontró una asociación entre la presencia de atrofia glandular en la biopsia de la glándula salival menor y el desarrollo de poliautoinmunidad (OR = 11,1; IC_95%_: 1,12112; p = 0,033). En todas las muestras se encontró expresión de los cuatro biomarcadores (CD3, CD4, CD8 y CD20) mediante inmunohistoquímica.

**Cuadro 2:** Características histopatológicas de las biopsias de glándula salival menor

Inflamación (N = 42)	40/42 (95,2)
Leve	6 (14,3)
Moderada	29 (69)
Severa	5 (11,9)
Dilatación ductal	33/42 (78,6)
Fibrosis	30/42 (71,4)
Atrofia	30/42 (71,4)
Adiposis	8/40 (19)
Centros germinales	1/42 (2,4)
Granulomas	0/42 (0)
**Puntaje de foco (N = 42)**	
Mediana (RIC)	1,4 (0,7)
1 a 2	36 (85,7)
2,1 a 3	5 (11,9)
≥ 3,1	1 (2,4)

RIC: rango intercuartílico

Por otro lado, cuando se compararon los pacientes con síndrome de Sjögren y otras enfermedades autoinmunitarias respecto a los hallazgos inmunohistoquímicos de las biopsias de las glándulas salivales menores, la mayoría presentaron relaciones de CD20:CD3 y de CD4:CD8 ≤ 2:1, sin diferencias estadísticamente significativas entre los dos grupos (p > 0,05). En el subgrupo de pacientes con síndrome de Sjögren, solo un caso presentó una relación CD4:CD8 de 1:1, ocho casos tuvieron una relación de 2:1 y los siete restantes, relaciones de 3:1 y 4:1. Estos 15 casos denotan una predominancia de linfocitos T CD4 en estas pacientes. De los casos analizados, la mayoría de las pacientes con síndrome de Sjögren presentaron inflamación glandular moderada (cuadro 3). Finalmente, seis pacientes con síndrome de Sjögren tenían datos suficientes para el cálculo del ESSDAI. En el cuadro suplementario 1 se presenta la distribución de acuerdo con el puntaje y la duración de la enfermedad según los grados de inflamación de la biopsia de glándula salival menor.

**Cuadro 3:** Hallazgos histopatológicos e inmunohistoquímicos de la biopsia de glándula salival menor según la enfermedad autoinmune, independientemente de si se presenta o no en pacientes con pacientes con poliautoinmunidad

Enfermedad autoinmunitaria	Puntaje de foco			Inflamación			CD20:CD3		CD4:CD8	
	1 - 2 n	> 2 n	Leve n	Moderada n	Grave n	2:1 n (%)	3:1 a 4:1 n (%)	1:1 a 2:1 n (%)	3:1 a 4:1 n (%)
Síndrome de Sjögren	13/16	3/16	3/16	10/16	3/16	12/16	4/16	9/16	7/16
Artritis reumatoide	6/7	1/7	-	7/7	-	5/7	2/7	3/7	1/7
Lupus eritematoso sistémico	6/6	-	-	5/6	-	5/6	1/6	4/6	2/6
Esclerosis sistémica	4/4	-	1/4	3/4	-	3/4	1/4	4/4	-
Síndrome antifosfolípido	1/1	-	-	1/1	-	1/1	-	1/1	-
Enfermedad mixta del tejido conjuntivo	-	1/1	-	1/1	-	1/1	-	1/1	-
Miopatía inflamatoria idiopática	1/1	-	-	1/1	-	1/1	-	1/1	-
Cirrosis biliar primaria	1/1	-	-	1/1	-	-	1/1	1/1	-

No hubo diferencias estadísticamente significativas (prueba exacta de Fisher: p > 0.05).

## Discusión

Este estudio planeó describir las características clínicas, histopatológicas e inmunohistoquímicas (poblaciones linfocitarias T y B) de los pacientes con síntomas secos -con síndrome de Sjögren o no- o que padecieran alguna enfermedad reumatológica autoinmunitaria sistémica. A estas pacientes se les practicó biopsia de glándula salival menor para hacer el diagnóstico diferencial de síndrome de Sjögren y establecer relaciones con las variables inmunohistoquímicas, poco documentadas en la literatura.

El presente estudio encontró una mayor frecuencia de síndrome seco en mujeres en la quinta década de la vida similar a lo descrito por Moreno- Quispe *et al*. en la población peruana ^16^.

Respecto a las manifestaciones extraglandulares o sistémicas, el compromiso articular fue el síntoma más común en estas pacientes. Este hallazgo es similar a estudios previos que reportan un mayor compromiso articular no erosivo, particularmente en el subgrupo de pacientes con síndrome de Sjögren confirmado (n = 16) ^17^. En este último subgrupo, la poliautoinmunidad se presentó en la mitad de los casos (n = 8), similar a lo reportado por Lockshin *et al*., quienes registraron una frecuencia de síndrome de Sjögren acompañado por otra enfermedad autoinmune en el 52 % de los casos ^18^. El lupus eritematoso sistémico, la artritis reumatoide y la esclerosis sistémica fueron las enfermedades más frecuentes en la población evaluada con poliautoinmunidad. Estos hallazgos concuerdan con los publicados en el metaanálisis de Alani *et al*., pues la prevalencia de síndrome de Sjögren y lupus eritematoso sistémico fue del 19 % de los pacientes y la de síndrome de Sjögren y artritis reumatoide fue del 14 % de los pacientes ^19^.

Respecto al puntaje de foco, no hubo diferencias entre las pacientes con síndrome de Sjögren que tenían manifestaciones glandulares graves y aquellas con manifestaciones extraglandulares sistémicas. Sin embargo, la mayoría de los pacientes presentaron un puntaje de foco de dos o menos. Esto contrasta con lo descrito en otros reportes ^20^, en los que algunos pacientes desarrollan manifestaciones sistémicas de manera tardía, especialmente cuando tienen un puntaje de foco de tres. Lo mencionado se alinea con los hallazgos de Dal Pozzolo *et al*. ^21^, quienes resaltan que la caracterización patológica de las glándulas salivales y los valores altos del puntaje de foco no solo son útiles para el diagnóstico o la clasificación del síndrome de Sjögren, sino que proporcionan información para estratificar la gravedad de la enfermedad y predecir manifestaciones sistémicas, incluyendo su progresión a linfoma. De esta forma, el puntaje de foco podría considerarse un marcador histopatológico para el desarrollo de linfoma ^22^.

Por otro lado, se encontró una mayor frecuencia de atrofia en la biopsia de glándula salival menor en los casos de poliautoinmunidad, aunque en reportes similares no se han descrito estos hallazgos. Sin embargo, la presencia de atrofia es un factor interesante, dada la controversia respecto a la interpretación de la biopsia de la glándula salival menor y la inclusión de atrofia acinar, fibrosis y reemplazo graso.

Según el consenso de Fisher *et al*. ^6^, se recomienda informar el grado (ausente, leve, moderado o grave) de atrofia, fibrosis, dilatación de los conductos y sialoadenitis crónica inespecífica; también se debe mencionar la presencia o ausencia de sialoadenitis focal linfocítica. No obstante, esta última no se puede describir si la apariencia histológica de las glándulas está dominada por características asociadas con la sialoadenitis crónica inespecífica, como atrofia acinar, dilatación de los conductos y fibrosis, sin evidencia de focos adyacentes al parénquima normal. Por el contrario, si hay sialoadenitis crónica inespecífica, puede haber algunos focos en el síndrome de Sjögren adyacentes a áreas atróficas. La recomendación de los expertos es que la extensión de los cambios atróficos debe clasificarse e informarse para ayudar al clínico con su interpretación ^6^^,^^23^^,^^24^. De hecho, en el presente estudio, se encontró la misma frecuencia de atrofia y fibrosis en todos los casos, lo cual concuerda con lo descrito por Leehan *et al*. ^23^, quienes reportaron que los pacientes con síndrome de Sjögren tienen una presencia significativamente mayor de tejido fibrótico en la biopsia de glándula salival menor en comparación con aquellos que no sufren de síndrome de Sjögren.

En relación con la inmunohistoquímica, son pocos los estudios que analizan esta técnica en la biopsia de glándula salival menor y casi nulos los realizados en poblaciones colombianas o latinoamericanas, por lo cual los hallazgos del presente estudio son novedosos. En una investigación previa, se encontró que la inmunorreactividad de CD20 (marcador de linfocitos B) fue estadísticamente mayor en biopsias de glándulas salivales menores de 15 pacientes con síndrome de Sjögren en comparación con aquellos con sarcoidosis; el CD8 (marcador de linfocitos T citotóxicos) no mostró ninguna diferencia. Si bien en el presente estudio no se comparó la población objeto con otro tipo de pacientes, la presencia de CD20 y CD8 se observó en todas de las muestras ^25^. Además, en otra cohorte que incluyó 71 pacientes con síndrome de Sjögren, se detectó CD20 en muestras de glándulas labiales de todos los participantes, y el CD3 (linfocitos T) fue positivo solo en 66 (93,0 %) de las muestras ^26^. Sin embargo, en el presente estudio, todas las biopsias de las pacientes expresaron CD3.

Cabe resaltar que las pacientes analizadas con síndrome de Sjögren no tenían enfermedad tardía y presentaron hallazgos predominantemente de CD4 (marcador de linfocitos T ayudadores). Esto se podría relacionar con los hallazgos de un estudio previo que reportó predominio de linfocitos Th17 CD4 en muestras periféricas de pacientes con síndrome de Sjögren temprano ^27^.

En otro reporte, de una cohorte de 40 pacientes con síndrome de Sjögren ^28^, se determinó que el inmunofenotipo de muestras de saliva de glándulas parótidas (según los autores, semejante al de las biopsias glandulares) tenía un promedio de 71,7 % de células T (CD3^+^), 41,6 % de células T colaboradoras (CD3^+^/CD4^+^) y 53 % de células T citotóxicas (CD3^+^/CD8^+^). Por el contrario, todas las muestras de las pacientes de este estudio expresaron los clústeres de diferenciación seleccionados, con predominancia de las razones CD20:CD3 y CD4:CD8 sin mayores diferencias entre los pacientes con síndrome de Sjögren respecto a aquellos con otras enfermedades reumatológicas autoinmunes sistémicas ^28^.

Este hallazgo también fue documentado por Ono *et al*. ^29^, quienes encontraron más células T CD4 positivas que CD8 positivas entre los linfocitos infiltrantes de 100 biopsias de glándulas salivales menores, obtenidas de 20 mujeres con síndrome de Sjögren primario (cinco muestras por paciente) ^30^.

Los hallazgos histopatológicos de las biopsias de glándulas salivales menores y los valores del puntaje de foco siguen siendo componentes críticos para el diagnóstico del síndrome de Sjögren, ya que son elementos diferenciales del síndrome seco en pacientes con diferentes enfermedades reumatológicas autoinmunitarias sistémicas. Sin embargo, cabe resaltar que el puntaje de foco tiene una sensibilidad variable con diferentes limitaciones, tales como la falta de suficientes lóbulos de las glándulas salivales o área de superficie en la muestra, variabilidad de la distribución linfocítica en los acinos salivales, discrepancia en la interpretación de la biopsia de glándula salival menor, y la dificultad y la variabilidad de la evaluación de los infiltrados linfocíticos utilizando solo hematoxilina y eosina. Respecto a este último punto, se ha considerado que agregar tinciones de inmunohistoquímica para marcadores linfoides puede ayudar a evaluar estos infiltrados con mayor precisión.

Los marcadores CD3 y CD20 son específicos de linfocitos T y B, respectivamente. Por esta razón, se estableció que el uso de tinciones inmunohistoquímicas dirigidas contra estos marcadores en la biopsia de glándula salival menor destacaría los focos linfocíticos y aumentaría el área que cumpliera con los criterios histológicos para pacientes con síndrome de Sjögren. Esta propuesta está sustentada por los hallazgos de Trivedi *et al*.^31^, donde 35 de 45 pacientes fueron diagnosticados con síndrome de Sjögren según los criterios ACR/EULAR 2016. Al teñir las biopsias de glándulas salivales menores con hematoxilina-eosina, sólo 22 de los 35 casos cumplían los criterios histológicos para el diagnóstico de síndrome de Sjögren, pero luego de agregar la tinción inmunohistoquímica contra CD45, CD3 y CD20, el número incrementó a 29 casos ^31^. Sobre el uso de la inmunohistoquímica aún quedan algunos puntos de incertidumbre que deben continuar en estudio para validar su utilidad en el acercamiento diagnóstico y pronóstico del síndrome de Sjögren, como la afectación de su rendimiento por el uso de terapias inmunosupresoras previas.

Las limitaciones de este estudio incluyen el tamaño reducido de la muestra y de los subgrupos de pacientes. No fue posible concluir si las cinco pacientes sin síndrome de Sjögren o enfermedad reumatológica autoinmunitaria sistémica, pero con puntaje de foco positivo, no presentaban autoinmunidad, ya que no se dispuso en todos los casos de pruebas oftalmológicas objetivas como el puntaje de tinción ocular.

De manera similar y debido a la falta de datos -por el carácter retrospectivo del estudio- de los pacientes con enfermedad reumatológica autoinmunitaria sistémica, sin poliautoinmunidad con síndrome de Sjögren, y puntaje de foco positivo, no se pudo comprobar el síndrome de Sjögren. Además, la duración de los síntomas secos en estos pacientes (dos años o menos) es menor que la descrita en otros estudios, lo que plantea dudas sobre si se trataba de casos de síndrome de Sjögren en sus fases iniciales.

También se debe considerar que la naturaleza retrospectiva del estudio limitó la recolección de algunas variables. Aunque las historias clínicas no mencionaban otras enfermedades que deben excluirse antes de diagnosticar el síndrome de Sjögren según los criterios ACR/EULAR 2016 ^5^, no se contó con todas las variables específicas necesarias para afirmarlo.

Otra limitación fue la falta de análisis de los tratamientos inmunosupresores, que si bien no era uno de los objetivos del estudio, podría influir en los resultados de la inmunohistoquímica, particularmente en aquellos del subgrupo de pacientes con síndrome de Sjögren o con alguna enfermedad reumatológica autoinmunitaria sistémica. Sin embargo, este punto puede ser importante para analizar en el futuro en otros estudios.

En conclusión, este es el primer estudio de una población colombiana que evalúa el síndrome seco mediante el perfil inmunohistoquímico de la biopsia de glándula salival menor y que, además, describe las características sociodemográficas y clínicas de los pacientes. Se destaca que aquellos pacientes con un puntaje de foco de uno o más tenían expresión de los cuatro biomarcadores evaluados (CD3, CD4, CD8 y CD20). Se subraya la necesidad de realizar futuros estudios con valores menores en el puntaje de foco para evaluar de manera objetiva la utilidad de la inmunohistoquímica en la sensibilidad y en la precisión diagnóstica de la biopsia de la glándula salival menor. Además, se recomienda contar con un mayor tamaño de muestra y diferenciar otras causas de sequedad, especialmente en el subgrupo de pacientes con enfermedades reumatológicas autoinmunitarias sistémicas.

El predominio de linfocitos CD4 positivos en el análisis de inmunohistoquímica del subgrupo de pacientes con síndrome de Sjögren es considerable y ha sido demostrado por otros investigadores. Por esta razón, CD4 podría postularse como un biomarcador de aparición temprana de la enfermedad. Sin embargo, debido a las limitaciones del estudio, los resultados actuales no permiten establecer esta conclusión de manera contundente.

No obstante, según los resultados de este estudio, se considera que la implementación de la inmunohistoquímica podría mejorar la visualización de los hallazgos de la biopsia de glándula salival menor. Esto facilitaría el diagnóstico de los pacientes con síndrome de Sjögren y contribuiría al diagnóstico diferencial. Además, podría aportar a la estratificación del riesgo, la evaluación de la actividad de la enfermedad, el pronóstico y la identificación de nuevas dianas terapéuticas. La integración de los perfiles inmunohistoquímicos de los marcadores evaluados en el presente estudio, y otros no evaluados, podría ayudar a desarrollar estrategias de tratamiento más efectivas para el síndrome de Sjögren dada la escasez de opciones terapéuticas actuales para esta enfermedad. Por todo lo anterior, se requieren estudios con mayor solidez metodológica que evalúen otros marcadores mediante inmunohistoquímica en biopsias de glándula salival menor.

## Archivos suplementarios

**Cuadro suplementario:** 1. Distribución entre la duración de la enfermedad y el puntaje EULAR *Sjögren's syndrome disease activity index* (ESSDAI) según las características histopatológicas de la biopsia de glándula salival menor en pacientes con síndrome de Sjögren. (N = 16)

		Puntaje de foco		Inflamación			CD20:CD3		CD4:CD8	
		1-2	≥2,1	Leve n	Moderada n	Grave n	2:1	3:1 a 4:1	1:1 a 2:1	3:1 a 4:1
Duración de la enfermedad (años), n										
	0	2	2	-	2	2	4	-	1	3
	1	3	-	2	1	-	1	2	2	1
	2	2	-	-	2	-	2	-	1	1
	3	2	1	-	2	1	1	2	2	1
	> 4	1	-	-	1	-	1	-	1	-
ESSDAI, actividad, n										
	Leve	3	1	1	1	2	3	1	1	3
	Moderada	1	-	1	-	-	1	-	1	-
	Grave	1	-	-	1	-	1	-	1	-

No hubo diferencias estadísticamente significativas (prueba exacta de Fisher, p > 0.05).
